# Oxidative Stress Markers and Antioxidant Enzymes in Children and Adolescents with Depressive Disorder and Impact of Omega-3 Fatty Acids in Randomised Clinical Trial

**DOI:** 10.3390/antiox10081256

**Published:** 2021-08-05

**Authors:** Barbora Katrenčíková, Magdaléna Vaváková, Zuzana Paduchová, Zuzana Nagyová, Iveta Garaiova, Jana Muchová, Zdenka Ďuračková, Jana Trebatická

**Affiliations:** 1 Faculty of Medicine, Institute of Medical Chemistry, Biochemistry and Clinical Biochemistry, Comenius University, Sasinkova 2, 813 72 Bratislava, Slovakia; katrencikov2@uniba.sk (B.K.); magdalena.vavakova@med.lu.se (M.V.); zuzana.paduchova@fmed.uniba.sk (Z.P.); jana.muchova@fmed.uniba.sk (J.M.); 2 Juvenalia, s.r.o., Paediatric Centre, Veľkoblahovská 44A, 929 01 Dunajská Streda, Slovakia; juvenaliads@gmail.com; 3 Research and Development Department, Cultech Ltd., Unit 2 Christchurch Road, Port Talbot SA12 7BZ, UK; ivetag@cultech.co.uk; 4 Department of Paediatric Psychiatry, Faculty of Medicine, The National Institute of Children’s Diseases, Comenius University, Limbová 1, 833 40 Bratislava, Slovakia; jana.trebaticka@fmed.uniba.sk

**Keywords:** depressive disorder, omega-3 fatty acids, oxidative stress, lipoperoxides, 8-isoprostane, advanced oxidative protein products, nitrotyrosine, antioxidant enzymes, children and adolescents

## Abstract

Oxidative stress (OS) is thought to play a role in mental disorders. However, it is not clear whether the OS is the cause or consequence of the disorder. We investigated markers of oxidative stress (8-isoprostane (8-IsoP-U), lipoperoxides (LP), advanced oxidation protein products (AOPP) and nitrotyrosine (NT)) and antioxidant protection (Trolox equivalent antioxidant capacity (TEAC), activities of superoxide dismutase (SOD), glutathione peroxidase (GPx) and catalase (CAT) in 60 paediatric and adolescent patients with depressive disorder (DD) compared to healthy controls. The patients were divided into two groups (1:1). One group received an emulsion of omega-3 fatty acid (FA), and the other group an emulsion of sunflower oil with omega-6 FA for 12 weeks. The levels of 8-IsoP-U, AOPP and NT were increased, and GPx activity was decreased in patients compared to the controls. We found a significant positive correlation of the Children’s Depression Inventory score with NT and a negative correlation with TEAC, SOD and GPx. NT correlated positively with the baseline omega-6/omega-3 FA ratio and a negatively with SOD. A supplementation with omega-3 FA, but not with omega-6 FA, decreased 8-IsoP-U, AOPP, NT levels and increased TEAC and SOD activity. Our results suggest that NT may play a role in the pathophysiology of DD, while elevated isoprostane is likely caused by the high omega-6/omega-3 FA ratio. Omega-3 FA supplementation reduces oxidative stress in patients with DD. This study was registered with the ISRCTN registry (ISRCTN81655012).

## 1. Introduction

Oxidative stress (OS) is a phenomenon that is associated with many diseases [1]. It remains to be elucidated whether oxidative stress is the cause or the consequence of pathological conditions [2,3]. In some diseases, an increased production of reactive oxygen (ROS) and nitrogen (RNS) species can damage some organs and can be one of the causes of the disease (e.g., type I diabetes) [4]. In other diseases, oxidative stress occurs as consequence of a pathological condition [5]. Psychological stress also significantly contributes to OS [6]. Oxidative stress has long been thought to play a role in neurodegenerative diseases, such as Alzheimer’s disease, Huntington’s disease, and Parkinson’s disease. At present, it is assumed that OS is involved in the pathophysiology of neuropsychiatric diseases such as anxiety disorders, attention deficit hyperactivity disorder (ADHD), schizophrenia and depression disorders (DD) [7,8,9,10,11]. The presumption of oxidative stress involved in psychiatric disorders is supported by the fact that the neurons in the brain have high levels of phospholipids and polyunsaturated higher carboxylic acids sensitive to oxidation, high oxygen levels and energy consumption, as well as low levels of antioxidants [9].

Altered levels of OS markers were confirmed, e.g., in schizophrenia [11]. Low levels of antioxidant enzymes, Cu/Zn superoxide dismutase (SOD) and glutathione peroxidase (GPx) were observed in patients with a panic disorder [12] and affective disorders, including depressive disorder in adults [13].

Increased markers of oxidative stress (lipid hydroperoxides, 8-isoprostane and protein carbonyls) or decreased levels of antioxidants were reported in the meta-analysis of Black et al. [14] and in several other studies in adults with depressive disorder in human [15,16] or in animal models [17].

However, there is only sporadic information on the relationship between oxidative stress and depressive disorders in children and adolescents.

Freed et al. [18] tested the level of reduced glutathione (GSH), an important marker of the redox state of organisms, in the brains of adolescents with depressive disorders using proton magnetic resonance spectroscopy. They found the lower levels of GSH in 19 depressed patients compared to the controls, although there was no correlation with the severity of depression or its onset. Magalhães et al. [19] showed that young adults with early stages of bipolar disorder (BD) or depressive disorder had higher protein carbonyl levels compared to the controls. On the contrary, Scola et al. [20] reported no differences in the protein carbonyl levels of BD controls. In contrast to the findings in adults [14], the levels of lipoperoxides were lower in adolescents, with varying risk for the development of BD. The authors found that the level of lipoperoxides (early-stage lipid peroxidation marker) was lower in patients with first-episode of bipolar disorder compared to the controls. Moreover, the severity of depression was negatively correlated with lipoperoxides, while the severity of manic symptoms correlated with lipoperoxides positively [20].

Despite the inconsistency in the results regarding markers of oxidative stress, especially oxidative damage to lipids, the inhibition of oxidative damage to biologically important molecules may reduce disorder progression and severity [21].

Polyunsaturated fatty acids are involved in the regulation of inflammation through their metabolites, as well as due to their conformation, which may influence the fluidity of membrane. By these mechanisms, FAs can interfere with the pathophysiology of DD. Arachidonic acid (AA, C20:4, ω = 6) is a substrate for the synthesis of eicosanoids, stimulating the synthesis of proinflammatory cytokines by cyclooxygenase (COX). On the other hand, omega-3 fatty acids, eicosapentaenoic acid (EPA, C20:5, ω = 3) and docosahexaenoic acid (DHA, C22:6, ω = 3) are converted to D-resolvins or E-resolvins, both contributing to the anti-inflammatory properties. Due to the formation of metabolites with opposite properties (pro-inflammatory from AA and anti-inflammatory from EPA and DHA), the omega-6/omega-3 FA ratio plays an important role in the regulation of a pro-/anti-inflammatory balance [22]. An increased omega-6/omega-3 FA ratio follows the substitution of DHA by arachidonic acid in the brain, leading to an increase in the turnover of AA to proinflammatory metabolites [23].

In our recent study [24], we observed that children with depressive symptoms have a higher serum ratio of omega-6/omega-3 FA compared to healthy children. The ratio of omega-6/omega-3 was reduced in DD patients following three months of omega-3 FA supplementation.

The outcome results demonstrated in present work are part of the project DEPOXIN (Molecular basis of depression disorder and influence of omega-3 fatty acid on clinical symptoms and biomarkers, ISRCTN81655012). The primary outcomes evaluated the effects of omega-3 fatty acids on the severity of depressive symptoms in children and adolescents [24]. The secondary outcomes, in addition to those presented in this work, included monitoring of the omega-6/omega-3 FAs ratio [24], lipid parameters [25] and indirect markers of inflammation, such as thromboxane B, homocysteine or brain-derived neurotrophic factor (BDNF) and vitamin D [26] in relation to depression severity and the possible effect of omega-3 FA intervention.

In this work, we hypothesised whether oxidative stress will be associated with the severity of depression in children and adolescents and that omega-3 fatty acid supplementation may influence the markers of oxidative stress. The markers of oxidative stress include 8-isoprostane in urine (8-isoP-U) and lipoperoxides (LP) as markers of lipids, advanced oxidation protein products (AOPP) and nitrotyrosine (NT) as markers of proteins damage. In addition, the markers of antioxidant defence, such as the Trolox equivalent antioxidant capacity of serum (TEAC), Cu/Zn superoxide dismutase (SOD), glutathione peroxidase (GPx) and catalase (CAT) enzymes activities, were investigated.

## 2. Materials and Methods

A detailed description of the study design, enrolment of patients and their treatments were given in previous publications from the DEPOXIN project [24,25,26].

Briefly, we enrolled 60 patients aged 7–18 years with depressive disorder (DD) and mixed anxiety and depressive disorders (MADD), diagnosed according to the ICD-10 ((International Classification of Diseases) (Supplementary Figure S1) and 20 healthy controls of comparable ages and sex.

The exclusion criteria were chronic somatic diseases (endocrine, metabolic and autoimmune); dietary restrictions (vegetarians, lactose intolerance and celiac disease); psychotic disorders; eating disorders; an addiction to psychoactive compounds; personality disorders; organic mental disorders and pervasive developmental disorders.

We randomly divided the patients into two groups (1:1). The patients were supplemented with either emulsions of omega-3 FA-enriched fish oil (Om3 group) or with emulsions of omega-6 FA-rich sunflower oil (Om6 group) during the 12-week intervention period, in addition to their standard treatment with selective serotonin reuptake inhibitors (SSRIs).

The number of patients to be enrolled in the project was estimated using the statistical program StatsDirect for sample size as a randomised intervention study by the *t*-test, with the choice of parameters: estimated effect (difference between means or effect size), the decrease of the CDI score by 5 points, estimation of the population deviation (standard deviation) as 6, balanced design as 1:1, the test power at 80% and the Alpha level at 5%. The number of patients in each group (control and intervention) was estimated at 19. We recruited 60 patients altogether to account for potential dropouts. The patients who completed the 12 weeks of supplementation were included in the analysis.

The emulsion of fish oil consisted of 2.4 g of total omega-3 FA (1.0 g of EPA and 0.75 g of DHA, ratio EPA:DHA = 1.33:1), and the emulsion of sunflower oil contained 2.467 g of omega-6 linoleic acid (Cultech Ltd., Port Talbot, UK).

The patients were instructed to consume their usual diet without additional antioxidants and to inform the responsible physician of any dietary changes.

The severity of the depressive state was investigated and evaluated by a responsible psychiatrist using the self-rated scale of the Children’s Depression Inventory (CDI) [24]. A higher CDI score indicates a more severe depressive condition.

Patients’ parents signed informed consent, and, in addition, patients gave oral assent to participate in the study.

This study was approved by the Ethics Committee of the Faculty of Medicine, Comenius University and The National Institute of Children’s Diseases (20 March 2013).

### 2.1. Biochemical Parameters

Blood and urine were collected from patients at the baseline (week 0) and after 6 weeks (week 6) and 12 weeks of supplementation (week 12). Venous blood was collected from patients and healthy controls after a 12-h overnight fast. Serum and plasma were obtained by centrifuging the blood (1200× *g*, 10 min) with or without EDTA as an anticoagulant within 1 h of blood collection. To the remaining volume of whole blood with anticoagulant containing mostly erythrocytes, a saline solution (0.15-mol/L NaCl) was added, followed by centrifugation at 1200× *g*, 7 min, and the procedure was repeated 2 more times. The serum and plasma were aliquoted and stored at −80 °C until further use.

Washed erythrocytes were diluted 1:3 with chilled distilled water. After 15 min, the hemolysate was stored at −20 °C until further use. In the hemolysate, haemoglobin was determined by the Drabkin method [27] and expressed in g/L.

A second morning urine of the patients was collected (medium stream), aliquoted and stored at −20 °C until further use.

Urinary isoprostane were determined using the 8-Isoprostane ELISA Kit (Cayman Chemicals, No. 516351, Ann Arbor, 48108, MI, USA), according to the manufacturer’s protocols. Samples were diluted 25 times. The isoprostane concentration was given in ng/mmol creatinine.

The concentration of lipoperoxides was determined in the serum according to El-Saadani et al. [28]. The determination was based on the ability of peroxides to oxidatively convert iodide (I^−^) to iodine (I_2_). The iodine in the reaction mixture gradually reacted with an excess of iodide to form I_3_ with an absorption maximum of 365 nm. The concentration of lipoperoxides was given in nmol/mL.

The concentration of advanced protein oxidation products (AOPP) was determined at 340 nm, according to the method of Witko-Sarsat et al. [29], based on a calibration curve of chloramine T with potassium iodide. The AOPP concentration was given in µmol/L.

Plasma nitrotyrosine was determined by the Nitrotyrosine Elisa Kit (Hycult biotech, No. HK 501-02, 5405 PB, Uden, The Netherlands). The nitrotyrosine concentration was given in nmol/L.

The Trolox equivalent antioxidant capacity of serum (TEAC) assay measured the ability of antioxidants to scavenge the stable radical cation ABTS^+^ (2,2′-azinobis(3-ethylbenzothiazoline-6-sulfonic acid). A blue-green chromophore with maximum absorption at 734 nm was decolorised in the presence of both lipophilic and hydrophilic hydrogen-donating antioxidants. The antioxidant activity of the sample was compared with the antioxidant activity of the synthetic vitamin E, Trolox [30]. The concentration of Trolox equivalents was expressed in mmol/L.

SOD activity was determined in erythrocyte hemolysates using a kit from Randox, No. SD 125 RANSOD (Crumlin, Country Antrim, BT29 4QY, UK). Superoxide dismutase activity was reported in U/mg of haemoglobin (Hb).

Glutathione peroxidase activity was determined in erythrocyte hemolysates using the Glutathione Peroxidase Activity Kit (Enzo Life Sciences, No. ADI-900-158, CH-4415 Lausen, Switzerland, Biotech distributor), according to the manufacturer’s protocols. The activity was given in µkat/mg of Hb.

Catalase activity was determined in erythrocyte hemolysates, according to Bergmeyer [31]. The determination was based on the change in the absorbance of hydrogen peroxide over time at a wavelength of 240 nm. The activity was given in µkat/g of Hb.

Creatinine in the urine was determined at the Department of Clinical Biochemistry of the National Institute of Children’s Diseases using a Hitachi 911 analyser by a standard procedure using Roche Diagnostics kits (Roche Diagnostics International, Bratislava, Slovakia). The concentration was given in mmol/L.

### 2.2. Statistical Analysis

All data were presented as the mean ± standard deviation (SD) or as the median (Q1; Q3), according to the normality of data. The normality of data distribution was evaluated with a Shapiro–Wilk test. A Student’s *t*-test (8-IsoP-U, SOD and CAT) or Wilcoxon signed-rank test (LP, AOPP, NT, TEAC and GPx) were used to compare changes from the baseline in different timepoints. An unpaired *t*-test and a nonparametric Mann–Whitney *U* test were used, dependent on the normality of data for the evaluation of the differences between patients and healthy controls, two types of diagnoses and gender. The effect of the omega-3 fatty acids 12-week supplementation on the studied parameters was determined using a multifactorial ANOVA with age the covariate and diagnosis, gender, and supplement as the categorical variables, including the Bonferroni correction method. For the correlation determination, the Spearman’s rank correlation coefficient was used.

A level of *p* ≤ 0.05 was considered a statistically significant result. Statistical analyses of the data were performed with StatsDirect^®^ 3.3.4 (StatsDirect Ltd., Birkenhead, Merseyside CH42 8NQ, UK) and IBM SPSS Statistics 25.0.0 (New York 10504-1722, NY, USA). For the graphical representation of data, the StatsDirect^®^ program was used.

## 3. Results

In our patients, the 6 and 12 week supplementations with omega-3 FA, in contrast to omega-6-FA, reduced the severity of depression, as assessed by the CDI score by scores of 6.3 (22%) and 6.5 (24%), respectively. The omega-6/omega-3 ratio decreased from 24.2/1 to 7.6/1 after 6 weeks of omega-3 FA supplementation and after 12 weeks to 9.9/1 (69% and 60%, respectively). EPA negatively correlated with the severity of depression (CDI score), and the ratio of omega-6/omega-3 FA positively correlated with the CDI score. However, there was no correlation between the DHA and CDI scores [24].

### 3.1. Baseline Data

Detailed baseline characteristics, e.g., age, weight, height and BMI (kg/m^2^) of the patients with depressive disorder and healthy controls have been published [24,25,26], and the data are included in Supplementary Table S1.

Markers of oxidative stress, 8-isoP-U, LP, AOPP and NT in depressed patients and a healthy control group at the baseline are presented in Table 1. Significantly elevated levels of 8-IsoP-U, AOPP and NT were found in patients compared to the control group. Lipoperoxides did not differ from the controls (Table 1).

The marker of serum antioxidant capacity, TEAC, as well as the erythrocyte antioxidant enzyme activities SOD and CAT, were not found different in the patients compared to the controls at baseline. Decreased activity of the antioxidant enzyme GPx was observed in patients compared to the healthy controls (Table 1).

In depressed patients, we found a significant increase in 8-IsoP-U by 26%, AOPP by 92% and NT by 57% compared to the healthy control group. From antioxidant enzymes, only the activity of GPx in the patients was reduced by 19% compared to the controls.

### 3.2. Effect of FA Supplementation in Patients Suffering from Depressive Disorder 

The effect of omega-3 and omega-6 FA supplementation on the oxidative stress markers, TEAC and antioxidant enzyme activities are shown in Table 2.

The level of 8-IsoP-U decreased to 81% at 6 weeks and to 67% at 12 weeks from the baseline in the Om3 group. No significant changes in the 8-IsoP-U levels were observed in the Om6 group. LP was not affected by either omega-3 or omega-6 FA. The AOPP levels decreased to 86% at 6 weeks and to 91% at 12 weeks in comparison to the baseline after supplementation with omega-3 but not with omega-6 FA. NT was significantly affected by omega-3 FA at 6 weeks, but not at 12 weeks. NT levels were not changed in the omega-6 FA group (Table 2).

TEAC increased significantly in the Om3 group. Following the supplementation with omega-3 FA, but not omega-6 FA, SOD activity increased significantly from the baseline (106% at week 6 and 109% at week 12, respectively) (Table 2). The GPx and CAT activities were not affected by either omega-3 or omega-6 FA supplementation.

The significant difference between groups were observed in 8-IsoP-U levels (*p* = 0.029) and closed to a significant difference for SOD activity (*p* = 0.06) after 12 weeks of intervention (Table 2).

To clear the effect of the supplement from other variables (diagnosis, gender, and age), we used a multifactorial ANOVA with age as the covariate, including the Bonferroni correction method. For 8-IsoP-U, we showed a significant difference/effect of omega-3 FA (df (model) = 42, *p* = 0.0019; significant difference between Om3 and Om6 even after Bonferroni correction).

The Analysis of Variance including Bonferroni correction did not confirm a significant change in SOD activity following the omega-3 FA supplementation. 

### 3.3. The Correlations between Parameters

We examined the correlations between all the monitored parameters and the severity of depression characterised by the CDI score at the baseline, as well as the omega-6/omega-3 FA ratio [24] (Table 3).

Among the markers of oxidative stress, patients were found to have only a positive correlation between the NT and CDI. A negative correlation was confirmed between the CDI and TEAC, SOD or GPx. Similarly, we examined the correlations of the monitored markers with the omega-6/omega-3 FA ratio. The levels of 8-IsoP-U and NT showed positive correlations with the omega-6/omega-3 FA ratio, while the SOD activity correlated negatively with the omega-6/omega-3 ratio. In the healthy control, a positive correlation was only found between 8-IsoP-U and the omega-6/omega-3 FA ratio (Table 3).

Since our group of patients consisted of two different diagnoses (depressive disorder, DD subgroup, *n* = 35 and mixed anxiety and depressive disorder, MADD subgroup, *n* = 25), we examined the correlations between the CDI score and those markers of OS that were significant (Table 3) depending on the diagnosis.

**Figure 1:**
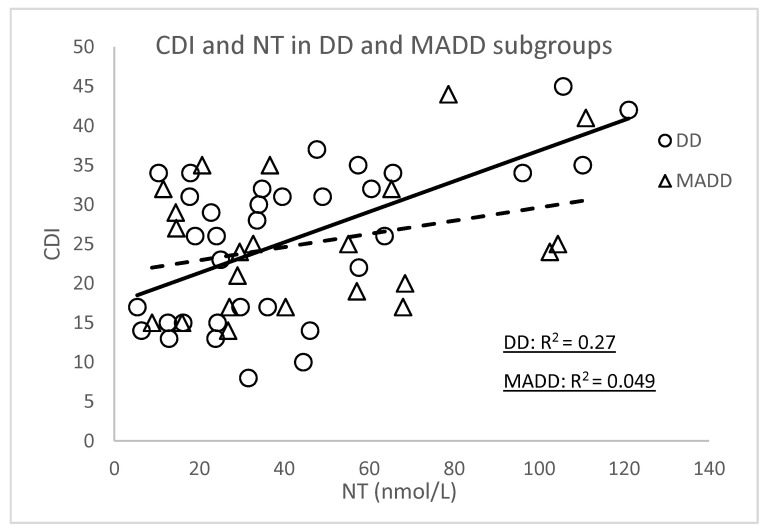

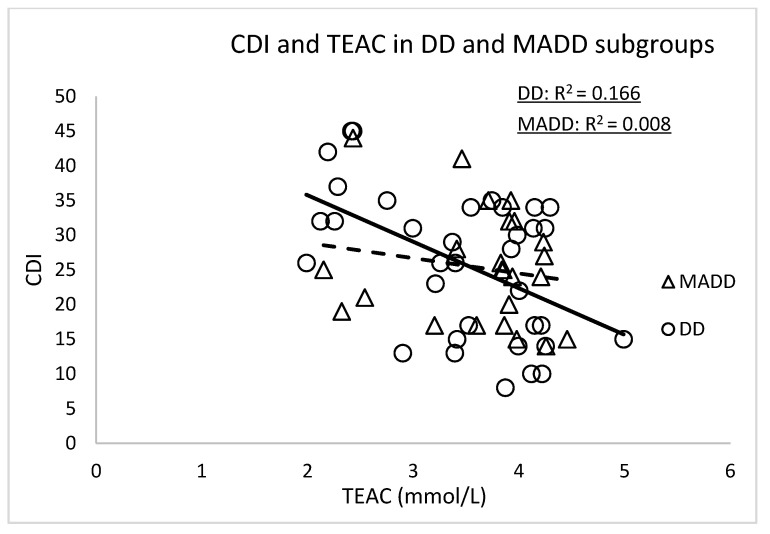
Correlations between the CDI versus NT and TEAC at the baseline in the DD and MADD subgroups. NT—nitrotyrosine, TEAC—Trolox equivalent antioxidant capacity, CDI—Children’s Depression Inventory, DD—depressive disorder subgroup and MADD—mixed anxiety and depressive disorder subgroup.

**Figure 2:**
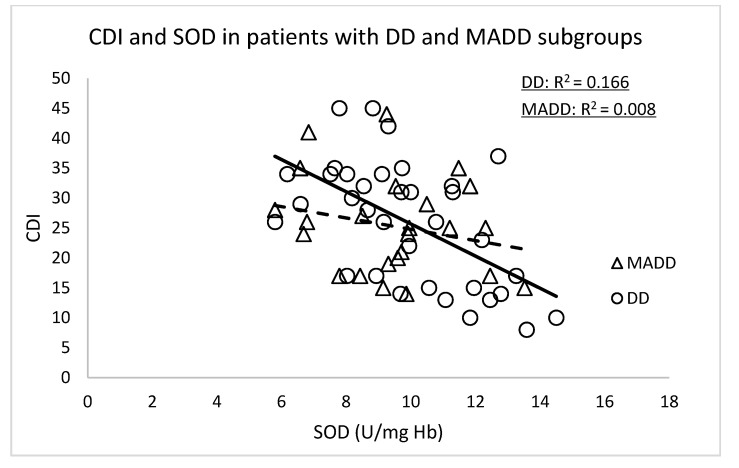

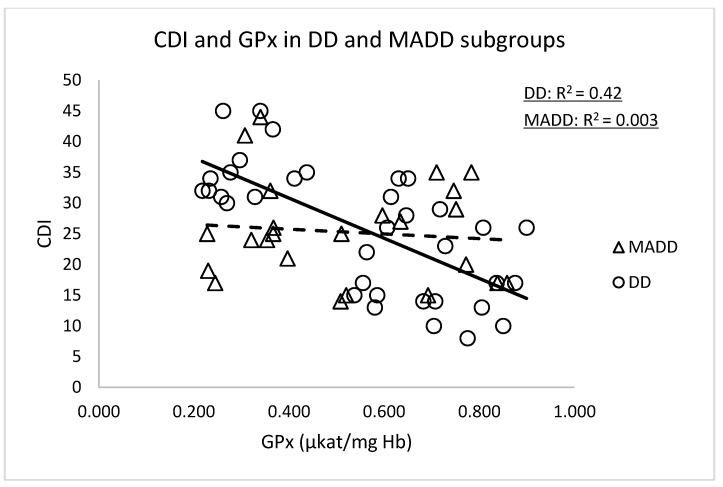
Correlations between the CDI versus SOD and GPx at the baseline in the DD and MADD subgroups. SOD—superoxide dismutase, GPx—glutathione peroxidase, CDI—Children’s Depressive Inventory, DD—depressive disorder subgroup and MADD—mixed anxiety and depressive disorder subgroup.

The significant correlations of the CDI score were confirmed in subgroup DD with NT (r = 0.520, *p* < 0.001), TEAC (r = −0.407, *p* = 0.024), SOD (r = −0.545, *p* < 0.001) and GPx (r = −0.648, *p* < 0.0001) (Figure 1 and Figure 2). In the group of patients with MADD, correlations between the CDI and the evaluated parameters were not observed.

Only NT levels correlated with the omega-6/omega-3 ratio in both subgroups (r = 0.414, *p* = 0.024 in the DD subgroup and r = 0.435, *p* = 0.024 in the MADD subgroup, respectively).

Significant correlations between TEAC, SOD or GPx and the omega-6/omega-3 ratio in the DD or MADD subgroups were not confirmed.

Following the omega-3 FA supplementation, no significant correlations were observed between the CDI or omega-6/omega-3 FAs and the monitored parameters after 6 weeks, except for the correlation between the CDI and SOD (r = −0.386, *p* = 0.019) and between the omega-6/omega-3 FA and NT (r = 0.475, *p* = 0.013).

In the Om6 group after 6 weeks of supplementation, a positive correlation with the CDI was observed for NT (r = 0.436, *p* = 0.012) and a negative correlation for GPx (−0.413, *p* = 0.015). TEAC was negatively correlated with the omega-6/omega-3 ratio (r = −0.370, *p* = 0.024).

## 4. Discussion

Fifty-eight paediatric and adolescent patients with depressive disorder and 20 healthy controls were evaluated for oxidative stress markers (8-IsoP-U, LP, AOPP, NT, TEAC, SOD, GPx and CAT) and their correlations with the depression severity or the serum omega-6/omega-3 ratio. The impact of 12 weeks supplementation with omega-3 FA was compared to the effect of omega-6 FA intervention.

Mental disorders, including depressive disorder, are associated with impaired brain function, a significant reduction in neuronal and glial cells in cortico-limbic regions, which is associated with changes in neuronal plasticity. These changes can trigger a production of reactive metabolites, resulting in cell death and the atrophy of neuronal and glial brain cells [32].

Lipoperoxides (early-stage lipid peroxidation markers) or isoprostane (late-stage marker of peroxidation) are the frequently evaluated markers of oxidative stress in depressive adults [14]. In contrast to adults, in whom an increase in LP was found, in adolescents early-stage lipid peroxides were observed to be decreased when compared to the healthy controls [20]. We have not observed any difference between the controls and our depressed patients. However, the later products of lipoperoxidation, 8-IsoP-U, was significantly increased in our patients compared to the healthy group. A direct comparison between the levels of the oxidative stress markers analysed in biological fluids and in the CNS was, however, not reliable, as a correlation between the plasma and cerebrospinal fluid has not been confirmed [21].

Markers of oxidative protein damage in depressed patients have been analysed, to a small extent compared to lipoperoxides. Protein carbonyls have been found to be increased in DD and bipolar disorder (BD) patients compared to the controls [14]. Similarly, increased levels of advanced oxidation protein products (AOPP) have been observed in DD and BD patients. The authors hypothesised that increased oxidation is due to insufficient catalase activity [33]. Similarly, we found significantly higher levels of AOPP in our depressed patients compared to the controls. In line with Maes et al. [33], no differences in the catalase activity between the studied groups was reported in this study. Despite the elevated levels of 8-IsoP-U and AOPP in depressed patients, their levels did not correlate with the CDI. Interestingly, 8-IsoP-U levels positively correlated with the omega-6/omega-3 ratio in the patients and healthy controls.

Despite the recommended omega-6/omega-3 ratio 5/1, we determined this ratio of 24.2/1 in our cohort and 19.3/1 in the control group [24]. The higher ratio in the control group may result in a production of lipid peroxidation even in “healthy” organisms.

NT is a product of nitration mediated by peroxynitrite that is formed from nitric oxide and superoxide, which can be released from activated inflammatory cells [34]. The NT levels in our patients were higher compared to the controls. One of the main pathophysiological features of DD is inflammation. Both NT and an increased ratio of omega-6/omega-3, are associated with inflammation; therefore, it is not surprising that NT levels significantly correlated with the CDI and, the omega-6/omega-3 ratio. These correlations were not observed in healthy children.

The effect of omega-3 FA on the severity of depression (determined by the CDI score) and the omega-6/omega-3 FA ratio has been reported in our previous study [24]. In our recent work [26], we found increased levels of thromboxane in our cohort of depressed patients compared to a healthy control group, which correlated positively with the CDI score. Platelet activation may contribute to oxidative stress [35], which is in line with our results findings. We observed increased levels of 8-IsoP-U, AOPP and NT in depressed patients compared to the controls concurrent with the reduced activity of the antioxidant enzyme GPx. The activities of SOD and CAT, together with the total antioxidant capacity (TEAC) were not different from the healthy controls.

There have been studies conducted on adults with DD in which the SOD activity is decreased [36] and, in others, increased [37,38] compared to the controls. Since SOD and GPx form a tandem in the elimination of superoxide and, subsequently, hydrogen peroxide, this imbalance (if the SOD activity is increased and GPX is decreased) [37] can lead to the increased oxidative stress.

Although we did not observe the differences in the SOD and CAT activities or TEAC between depressive children and healthy controls, the significant negative correlations were found between the CDI and TEAC, SOD and GPx, respectively.

From our results, it is not clear whether oxidative stress is a cause or a consequence of the depressed state of the patients. However, due to the elevated NT levels in the patients compared to healthy children, and a strong positive correlation between the CDI score and the omega-6/omega-3 FA ratio [24] suggests that NT is likely to play a role in the pathophysiology of depressive disorders. Despite the elevated levels of 8-IsoP-U and AOPP in patients compared to the controls, no relationship was found between the severity of depression or the omega-6/omega-3 FA ratio. Therefore, it can be speculated that these increased markers can be rather a result of a secondary pathological condition than directly due to DD pathophysiology.

Wiener et al. [39] found gender differences in adult patients with depressive disorder in AOPP (higher levels in women) but not in thiobarbituric acid reactive substances (TBARS). After gender stratification, no association was observed between the markers of oxidative stress and depressive symptoms. However, in our paediatric and adolescent patients, there were no differences between the genders at any endpoints (data not shown).

Our results show that there is no significant difference in antioxidant defence in children with a depressive disorder and a healthy control group, except for GPx activity. However, the strong negative correlation of TEAC, SOD or GPx with the CDI score highlight the importance of the total antioxidant capacity and antioxidant enzymes activities in the pathophysiology of depressive disorders.

The group of our patients consisted of two subgroups (depressive disorder, DD subgroup and mixed anxiety and depressive disorder, MADD subgroup). In our previous work [24], we found that the DD subgroup was more sensitive to omega-3 FA intervention compared to the MADD subgroup. For this reason, we studied the correlations for the DD and MADD subgroups at the baseline separately. Significant correlations between the CDI and NT, TEAC, SOD or GPx were confirmed in the DD subgroup but not in the MADD subgroup. This is in line with the conclusion of Black et al. (2015), that the interpretation of the results is strongly dependent on a well-defined diagnosis [14].

However, our conclusions need to be considered with caution, as correlations between markers of lipid peroxidation in the plasma and cerebrospinal fluid have not been confirmed [21,40]. Markers of oxidative stress are age-dependent, and therefore, the findings in adults may not be confirmed in children and adolescents and vice versa [20]. In addition, the same marker may be suitable for one disease and may not be suitable for another [41].

Oxidative stress is influenced by number of factors. In addition to endogenous factors (inflammation, disorders in certain signaling pathways, the uncontrollable production of ROS by some enzyme systems, damaged mitochondria, etc.) and exogenous factors (smoking, diet, physical activity, pollutants, etc.), genetic factors or epigenetic changes also play an important role in regulating oxidative stress.

It is generally believed that genetic factors may affect depression through the modulation of mitochondrial DNA and miRNA [42]. Mitochondrial SOD polymorphism (SOD2) has been found to increase the risk of depression in the elderly [43]. On the other hand, the SOD2 polymorphism rs4880 has no effect on the antidepressant response and inflammatory markers [44]. The influence of the paraoxonase 1 (PON1) polymorphisms, which affect the formation of ROS/RNS and the immuno-inflammatory characteristics of depression, has also been discussed [45,46]. Similarly, the PON1 polymorphism has been associated with depression and anxiety in individuals who are sensitive to contact with chemicals of nontoxic concentrations [47].

We examined PON1 with lactonase and arylesterase activities in depressed children without investigating the polymorphisms in our recent work. There was no difference in the activities of both PON1s from the control group. Similarly, both activities were not affected by omega-3 or omega-6 FA supplementation. However, PON1 lactonase activity correlated negatively with the CDI score at the baseline [25]. 

The relationship between oxidative stress and omega-3 FA in psychiatric disorders is not well-characterised. It is known that the administration of 2.2 g/day omega-3 FA to patients with the first episode of schizophrenia for 6 months reduced the symptom severity, decreased 8-isoprostane levels, and increased the overall plasma antioxidant capacity [48]. We observed similar results in our cohort of depressed patients; a decrease in the severity of depression was assessed by the CDI score after 3 months administration of omega-3 FA (2.4 g/day) [24], a decrease in 8-IsoP-U and AOPP levels, and an increase in TEAC and SOD activity.

The mechanism of omega-3 FA action on oxidative stress is currently not fully understood. It is assumed that oxidative stress and inflammation can influence each other [49]. One mechanism of the antioxidant/anti-inflammation effect of omega-3 FA could be the formation of neuroprostanes from DHA, which increases the level of antioxidant heme oxygenase-1 at the transcriptional level [50]. On the other hand, EPA with its anti-inflammatory properties can help reduce oxidative stress in depressed patients by reducing vascular inflammation [17,51] or through the inhibition of the immune inflammatory pathways [52].

Our study has some limitations. We did not record food diaries. However, the patients were advised not to consume antioxidant supplements during the study. When evaluating the effects of omega-3 FA in comparison with omega-6 FA on the individual parameters, we did not use *p*-value corrections due to the multiple testing. However, we used a multifactorial ANOVA with age as the covariate and diagnosis, gender, and supplement as the categorical variables, including the Bonferroni correction method.

## 5. Conclusions

In conclusion, we showed that NT is marker of oxidative stress that may be primarily involved in the pathophysiology of DD in children and adolescents. The 8-IsoP-U might be related to the low levels of omega-3 FA and high levels of omega-6 FA in the patients and healthy controls and has no causal relationship to depressive disorders. An analysis of the total antioxidant status, the antioxidant enzymes activities and their correlations with CDI score confirmed their potential role in the pathophysiology of depressive disorders; however, their true role in the pathogenesis of DD requires further investigation. Finally, we showed that the supplementation of omega-3 FA could contribute to the decrease of some oxidative stress markers in depressed children and adolescents.

## Figures and Tables

**Table 1:** Markers of oxidative stress; 8-isoP-U; LP; AOPP and NT and antioxidant systems TEAC, SOD, GPx and CAT in depressed patients and a healthy control group at the baseline.

Parameter	Healthy Controls	Patients	*p*
	(*n* = 20)	(*n* = 58)	
8-IsoP-U (ng/mmol Cr)	104.8 ± 18.2	132.4 ± 41.9	**0.009** ^a^
LP (nmol/mL)	46.3 (32.6; 31.6)	38.5 (27.7; 57.9)	0.279 ^b^
AOPP (µmol/L)	40.5 (31.2; 46.6)	73.8 (44.1; 87.1)	**<0.0001** ^b^
NT (nmol/L)	21.7 (11.4; 36.6)	34.0 (20.6; 56.6)	**0.026** ^b^
TEAC (mmol/L)	3.71 (3.41; 4.16)	3.85 (3.05; 4.21)	0.367 ^b^
SOD (U/mg Hb)	9.81 ± 5.68	9.74 ± 2.09	0.206 ^a^
GPx (µkat/mg Hb)	0.69 (0.54; 0.75)	0.56 (0.34; 0.71)	**0.015** ^b^
CAT (µkat/g Hb)	4.47 ± 0.81	4.23 ± 1.20	0.421 ^a^

C—healthy controls, P—patients, 8-IsoP-U—8-isoprostane in urine, LP—lipoperoxides, AOPP—advanced oxidation protein products, NT—nitrotyrosine, TEAC—Trolox equivalent antioxidant capacity, SOD—superoxide dismutase, GPx—glutathione peroxidase, CAT—catalase, Cr—creatinine, Hb—haemoglobin, *p*—significance, ^a^—unpaired *t*-test and ^b^—Mann–Whitney *U* test.

**Table 2:** The effect of omega-3 and omega-6 FA on the oxidative stress markers, TEAC and antioxidant enzyme activities.

	Patients—Om3					Patients—Om6					*p* between Om3 and Om6		
Parameters	Week 0	Week 6	*p* between 0 and 6	Week 12	*p* between 0 and 12	Week 0	Week 6	*p* between 0 and 6	Week 12	*p* between 0 and 12	Week 0	Week 6	Week 12
*n*	29	29		26		29	28		26	26			
8-IsoP-U (ng/mmol Cr)	133.8 ± 36.1	109.1 ± 44.1	**0.005**	89.9 ± 37.8	**<0.0001**	123.2 ± 32.2	119.4 ± 40.1	0.509	113.6 ± 29.3	0.173	0.276	0.425	0.029
LP (nmol/L)	38.9 (28.3; 67.7)	39.6 (26.5; 53.9)	0.732	36.4 (22.6; 46.6)	0.156	35.4 (27.2; 55.1)	30.3 (26.6; 44.7)	*0.060*	35.8 (24.6; 44.7)	0.338	0.587	0.391	0.957
AOPP (mmol/L)	70.2 (59.8; 87.0)	60.9 (38.7; 74.8)	**<0.001**	64.4 (47.4; 75.1)	**0.016**	75.7 (44.5; 90.6)	63.6 (39.7; 83.3)	0.565	69.1 (47.7; 85.5)	0.441	0.898	0.481	0.346
NT (nmol/L)	34.7 (24.0; 60.5)	33.0 (21.0; 57.6)	**0.036**	30.8 (15.7; 43.4)	0.157	32.1 (18.7; 56.4)	34.0 (18.6; 55.3)	0.635	30.1 (16.9; 52.7)	0.217	0.74	0.874	0.982
TEAC (mmol/L)	3.85 (3.05; 4.20)	3.96 (3.64; 4.18)	**0.018**	3.88 (3.51; 4.26)	**0.044**	3.83 (3.40; 4.00)	3.84 (3.65; 4.15)	*0.074*	3.84 (3.51; 4.09)	0.227	0.711	0.513	0.432
SOD (U/mg Hb)	9.77 ± 2.08	10.36 ± 2.21	**0.022**	10.68 ± 2.50	**0.035**	9.70 ± 2.10	10.11 ± 2.10	0.493	9.48 ± 1.90	0.764	0.901	0.664	*0.06*
GPx (mkat/mg Hb)	0.47 (0.32; 0.67)	0.59 (0.38; 0.69)	0.104	0.63 (0.37; 0.77)	0.431	0.59 (0.37; 0.74)	0.56 (0.40; 0.70)	0.493	0.64 (0.40; 0.72)	0.897	0.224	0.852	0.592
CAT (mkat/g Hb)	4.37 ± 1.42	4.32 ± 1.15	0.836	4.68 ± 1.47	0.478	4.10 ± 0.94	4.18 ± 1.5	0.756	4.06 ± 1.66	0.897	0.398	0.681	0.155

Om3—omega-3 group, Om6—omega-6 group, 8-IsoP-U—8-isoprostane in urine, LP—lipoperoxide, AOPP—advanced oxidation protein products, NT—nitrotyrosine, TEAC—Trolox equivalent antioxidant capacity, SOD—superoxide dismutase, GPx—glutathione peroxidase, CAT—catalase, Cr—creatinine, Hb—haemoglobin and *p*—significance. *p*-values between weeks 0 and 6 and between weeks 0 and 12 were analysed by a paired *t*-test (for 8-IsoP-U, SOD and CAT) and by Wilcoxon’s signed-rank test (for LP, AOPP, NT, TEAC and GPx); *p*-values between the Om3 and Om6 groups were analysed by an unpaired *t*-test (for 8-IsoP-U, SOD and CAT) and by the Mann–Whitney *U* test (for LP, AOPP, NT, TEAC and GPx).

**Table 3:** Correlations between the parameters in the patients and controls.

**All Patients at the Week 0**	***n***	**r**	***p***
CDI and 8-IsoP-U	55	0.033	0.404
CDI and LP	52	−0.135	0.169
CDI and AOPP	58	−0.013	0.461
CDI and NT	56	0.407	**<0.001**
CDI and TEAC	59	−0.343	**0.004**
CDI and SOD	59	−0.436	**<0.001**
CDI and GPx	59	−0.42	**<0.001**
CDI and CAT	58	−0.143	0.142
**All patients at the week 0**	***n***	**r**	***p***
omega-6/omega-3 and 8-IsoP-U	56	0.392	**0.004**
omega-6/omega-3 and LP	52	0.026	0.429
omega-6/omega-3 and AOPP	55	0.043	0.379
omega-6/omega-3 and NT	52	0.385	**0.005**
omega-6/omega-3 and TEAC	55	0.052	0.352
omega-6/omega-3 and SOD	55	−0.237	**0.046**
omega-6/omega-3 and GPx	55	−0.176	0.198
omega-6/omega-3 and CAT	54	0.057	0.682
**Controls**	***n***	**r**	***p***
omega-6/omega-3 and 8-IsoP-U	17	0.559	**0.020**
omega-6/omega-3 and LP	19	0.035	0.445
omega-6/omega-3 and AOPP	20	−0.023	0.461
omega-6/omega-3 and NT	20	0.144	0.273
omega-6/omega-3 and TEAC	20	0.277	0.242
omega-6/omega-3 and SOD	20	−0.364	0.138
omega-6/omega-3 and GPx	18	0.042	0.963
omega-6/omega-3 and CAT	19	0.244	0.309

CDI—Children’s Depressive Inventory, 8-IsoP-U—8-isoprostane in urine, LP—lipoperoxide, AOPP—advanced oxidation protein products, NT—nitrotyrosine, TEAC—Trolox equivalent antioxidant capacity, SOD—superoxide dismutase, GPx—glutathione peroxidase, CAT—catalase, *n*—number of evaluated children, r—Spearman’s rank correlation coefficient and *p*—significance.

## Data Availability

The datasets generated and analysed during the current study are not publicly available due to ethical reason but are available from the corresponding author upon reasonable request.

## Supplementary Materials

The following are available online at https://www.mdpi.com/article/10.3390/antiox10081256/s1, Supplementary Figure S1: Consort Flow Diagram. Supplementary Table S1: Baseline characteristics of the depressed patients and healthy controls.
