# Supplementary material for: Oxidative Stress Markers and Antioxidant Enzymes in Children and Adolescents with Depressive Disorder and Impact of Omega-3 Fatty Acids in Randomised Clinical Trial
Source: Antioxidants (Basel). 2021 Aug 5;10(8):1256. doi: 10.3390/antiox10081256 (PMC8389273; doi:10.3390/antiox10081256)

CONSORT

TRANSPARENT REPORTING of TRIALS

CONSORT 2010 Flow Diagram

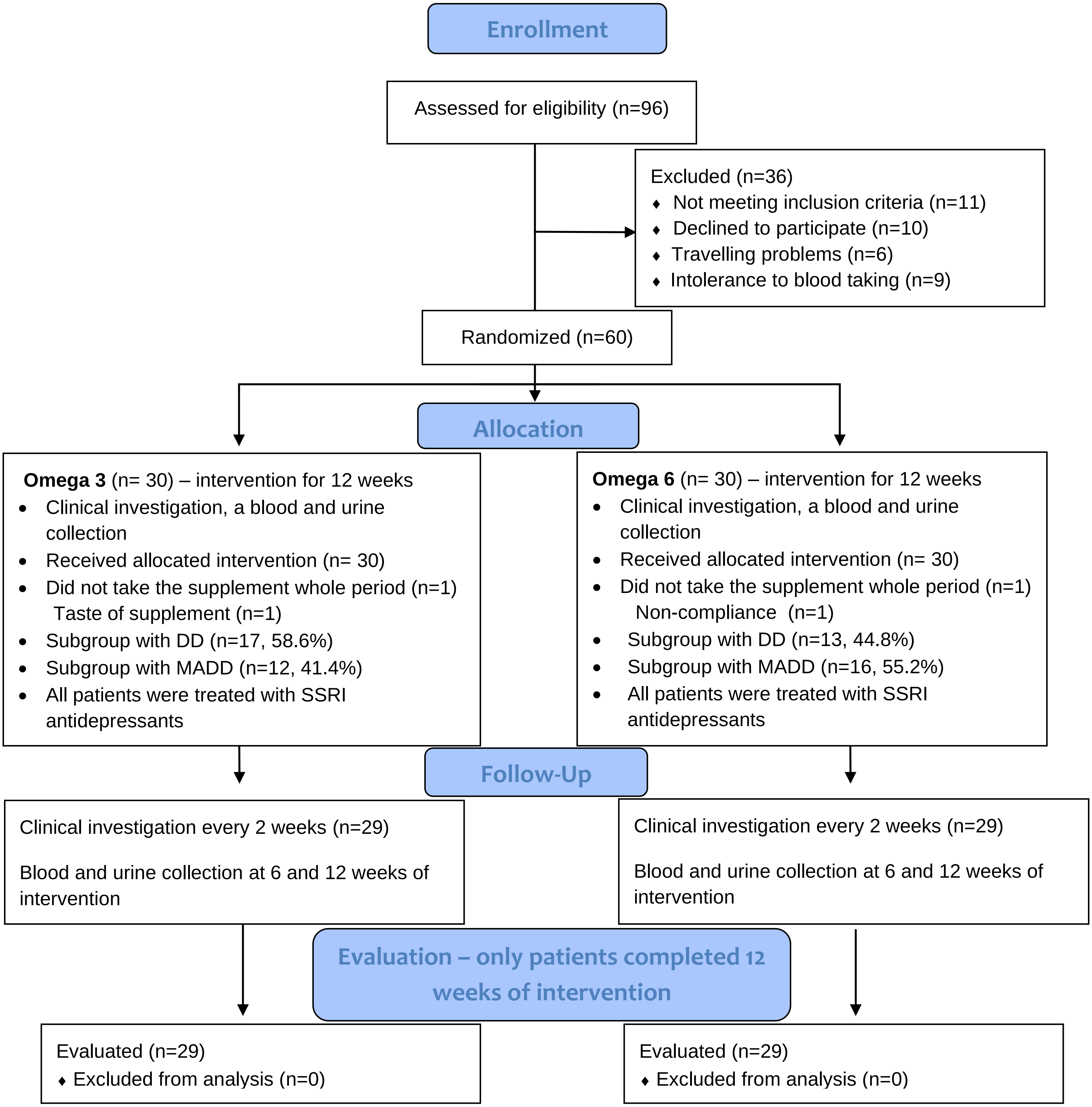

Supplement: Supplementary file 1 [file antioxidants-10-01256-s001.zip › Supplementary Figure S1.pdf]
