# Supplementary material for: Oxidative Stress Markers and Antioxidant Enzymes in Children and Adolescents with Depressive Disorder and Impact of Omega-3 Fatty Acids in Randomised Clinical Trial
Source: Antioxidants (Basel). 2021 Aug 5;10(8):1256. doi: 10.3390/antiox10081256 (PMC8389273; doi:10.3390/antiox10081256)
Supplement: Supplementary file 1 [file antioxidants-10-01256-s001.zip › Supplementary Table S1.pdf]

Table S1. Baseline characteristics of depressed patients and healthy controls.

Parameter	Patients			*p*-Value	Healthy Controls			*p*-Value	*p*-Value
	**All**	**Male**	**Female**	**M vs. F**	**All**	**Male**	**Female**	**M vs. F**	**P vs. C**
n	58	12	46		20	8	12		
Age (years)	15.6 ± 1.6	16.4 ± 2.2	15.3 ± 1.3	0.235	14.8 ± 2.4	14.0 ± 2.5	14.4 ± 2.5	0.621	0.059
Weight (kg)	60.3 ± 11.7	68.2 ± 15.5	56.8 ± 9.3	**0.015**	54.9 ± 18.8	56.1 ± 21.2	54.1 ± 17.8	0.435	0.147
Height (m)	1.68 ± 0.1	1.74 ± 01	1.66 ± 0.1	**0.016**	1.6 ± 0.2	1.6 ± 0.2	1.6 ± 0.1	0.543	0.471
BMI (kg/m2)	21.14 ± 2.7	22.4 ± 3.6	20.5 ± 2.8	0.532	20.6 ± 4.2	20.1 ± 3.0	20.9 ± 5.0	0.498	0.537

M = male, F = female, P = patient, C = healthy controls, *p* = statistical significance, vs. = versus, *n* = number of individuals.
